# Comparison of the Metabolic Profiles in the Plasma and Urine Samples Between Autistic and Typically Developing Boys: A Preliminary Study

**DOI:** 10.3389/fpsyt.2021.657105

**Published:** 2021-06-04

**Authors:** Xin-Jie Xu, Xiao-E Cai, Fan-Chao Meng, Tian-Jia Song, Xiao-Xi Wang, Yi-Zhen Wei, Fu-Jun Zhai, Bo Long, Jun Wang, Xin You, Rong Zhang

**Affiliations:** ^1^Medical Science Research Center, Research Center for Translational Medicine, Department of Scientific Research, Peking Union Medical College Hospital, Beijing, China; ^2^Key Laboratory for Neuroscience, Ministry of Education of China, Neuroscience Research Institute, Beijing, China; ^3^Key Laboratory for Neuroscience, National Committee of Health and Family Planning of China, Beijing, China; ^4^Department of Neurobiology, School of Basic Medical Sciences, Peking University, Beijing, China; ^5^Department of Rehabilitation Medicine, Beijing Tsinghua Changgung Hospital, Beijing, China; ^6^Department of Psychiatry, The Second Xiangya Hospital, Central South University, Changsha, China; ^7^Peking-Tsinghua Center for Life Sciences, School of Life Sciences, Beijing, China; ^8^Peking University McGovern Institute, Peking University, Beijing, China; ^9^Department of Education, Peking Union Medical College Hospital, Beijing, China; ^10^Department of Biomedicine and Biopharmacology, Hubei University of Technology, Wuhan, China; ^11^Department of Rheumatology and Clinical Immunology, Peking Union Medical College Hospital, Chinese Academy of Medical Sciences and Peking Union Medical College, Beijing, China

**Keywords:** autism spectrum disorder, metabolomics, metabolic profiling, plasma, urinary metabolites, taurine

## Abstract

**Background:** Autism spectrum disorder (ASD) is defined as a pervasive developmental disorder which is caused by genetic and environmental risk factors. Besides the core behavioral symptoms, accumulated results indicate children with ASD also share some metabolic abnormalities.

**Objectives:** To analyze the comprehensive metabolic profiles in both of the first-morning urine and plasma samples collected from the same cohort of autistic boys.

**Methods:** In this study, 30 autistic boys and 30 tightly matched healthy control (HC) boys (age range: 2.4~6.7 years) were recruited. First-morning urine and plasma samples were collected and the liquid chromatography–mass spectrometry (LC-MS) was applied to obtain the untargeted metabolic profiles. The acquired data were processed by multivariate analysis and the screened metabolites were grouped by metabolic pathway.

**Results:** Different discriminating metabolites were found in plasma and urine samples. Notably, taurine and catechol levels were decreased in urine but increased in plasma in the same cohort of ASD children. Enriched pathway analysis revealed that perturbations in taurine and hypotaurine metabolism, phenylalanine metabolism, and arginine and proline metabolism could be found in both of the plasma and urine samples.

**Conclusion:** These preliminary results suggest that a series of common metabolic perturbations exist in children with ASD, and confirmed the importance to have a comprehensive analysis of the metabolites in different biological samples to reveal the full picture of the complex metabolic patterns associated with ASD. Further targeted analyses are needed to validate these results in a larger cohort.

## Introduction

Autism spectrum disorder (ASD) is defined as a pervasive developmental disorder with lifelong symptoms manifested in the early postnatal period (1). Although the biological mechanisms are still not fully understood, results of recent studies suggest that genetic heritability, environmental risk factors and the interplay effects between them play important roles in the pathogenesis of ASD (2–4). The diagnosis of ASD is made mainly according to the children's behavioral symptoms, which includes impairments in communication and reciprocal social interaction, as well as restricted and repetitive behaviors and interests (5, 6).

In addition to the core symptoms of behavioral characteristics, accumulated results from recent studies indicate that children with ASD may also share some patterns of metabolic abnormalities (7–16). Metabolic alterations related to amino acids, carbohydrates and vitamins have been observed in ASD in previously studies (7–16). The untargeted metabolomics approaches offer a sensitive means to profile a wide range of metabolites, which will provide more information for further investigations of the disease mechanisms and to screen potential biomarkers for the diagnosis of ASD (7, 10, 14). These studies mainly used blood serum (16), plasma (7, 14, 15) or urine (8–13) samples separately, and the main methods used to perform metabolomic analysis were nuclear magnetic resonance (NMR) spectroscopy (10, 12, 15); liquid chromatography-mass spectrometry (LC-MS) (7, 14) and gas chromatography-mass spectrometry (GC-MS) (8, 9). Although some of the results from previous studies were consistent, there were also many inconsistent findings, with some of them even appearing contradictory. These inconsistencies may be related to the differences in participants' characteristics (ethnicity, age, and sex composition); sample types (urine, blood, or saliva) and methodologies (differences in technical methods in data acquisition, processing and analysis) chosen for the different studies. All these mentioned factors may be confounders which would cause bias and distort the association between the metabolites and ASD.

In order to eliminate the influence of these confounding factors, a total of 30 autistic boys and 30 tightly matched healthy control (HC) boys in a relatively narrow age range (2.4~6.7 years old) were recruited in this study. Only boys were recruited in this study to eliminate the verified sex difference associated in ASD (17–19). Moreover, both of the first-morning urine and plasma samples were collected and analyzed to obtain the comprehensive untargeted metabolic profiles. Results derived from different samples in the same cohort will significantly contribute to a fuller picture of the comprehensive and complex metabolic patterns associated with ASD.

## Materials and Methods

### Ethics Statement

This study was approved by the Peking University Institutional Review Board (IRB00001052-13079). Detailed information on the aims and protocols of the study were explained to the parents or legal guardians of the child participants. Written informed consent was obtained before the involvement of the child participants in the study.

### Participants

Autistic children were recruited from autism rehabilitation centers in Beijing, China (mainly from Wucailu Rehabilitation Center). The inclusion criteria for autistic children were: (1) Being diagnosed with autism which was confirmed by experienced psychiatrists according to the Diagnostic and Statistical Manual of Mental Disorders-IV-Text Revision (DSM-IV-TR, 2000) criteria. (2) Free of antibiotic treatment, prebiotics and probiotics for at least 4 weeks before sample collection. (3) The children's primary caregivers had good reading and comprehension skills and were able to fill in the relevant assessment scales. (4) The children's parents or legal guardians volunteered to participate in this study and signed the informed consent. Autistic children with symptoms of other comorbid neurological or psychiatric disorders were excluded from the study. Typically developing children in the control group were tightly matched with the ages of the autistic cases, and recruited through advertisements in kindergartens in Beijing. Children in the control group were also matched on the antibiotics, prebiotics, and probiotics criteria as well. Children were excluded from the control group if they have psychiatric conditions or other potentially confounding medical conditions.

### Assessment of Autistic Symptom

The following scales were used to assess autistic symptoms in autistic and typically developing children:

1. Childhood Autism Rating Scale (CARS): A 15-item behavior rating scale consists of 14 domains that are generally affected by severe autism, plus one category of general impressions of autism. It is widely used by psychiatrists during diagnosis of autism (20).
2. Autism Diagnostic Observation Schedule (ADOS): A semi-structured, standardized observation tool which is highly recognized as an evaluative measurement for diagnosing ASD. It includes a number of play-based activities designed to accurately assess and diagnose ASD based on the results found across the areas of communication, social interaction, play/imaginative use of materials, and restricted and repetitive behaviors (21).
3. Autism Diagnostic Interview-Revised (ADI-R): A standardized comprehensive interview which has proven highly useful for diagnosing autism and planning treatment. It provides categorical results for three domains: qualities of reciprocal social interaction; communication and language; and restricted and repetitive, stereotyped interests and behaviors. Together with the ADOS, it is recognized as the current gold standard of ASD diagnosis worldwide (22).
4. Gesell Developmental Schedules (GDS): A measure of child development which is designed to assess a child's neurodevelopmental status on the basis of the development quotient (DQ) scores in 4 domains: adaption, motor, language function, and personal/social function (23).

### Sample Collection

Blood samples were collected by trained nurses between 7:00 and 9:30 a.m. Parents were previously informed to have their children fast overnight and allowed only a moderate amount of drinking water to minimize the potential effects of food and water intake. Four milliliters of venous blood was collected into chilled EDTA tubes containing aprotinin (500 KIU/mL blood). Blood samples were centrifuged at 1,600 g for 15 min. Plasma was isolated and divided into 500 μL aliquots. The fresh first morning midstream urine was collected in sterile tubes. The samples were placed on ice and immediately frozen into dry ice and transferred to store at −80°C until assay.

### Sample Extraction

For plasma sample extraction, 200 μL of methanol was added to 100 μL of each thawed sample and vortexed for 60 s. The mixture was centrifuged at 12,000 rpm for 10 min at 4°C. For urine sample extraction, 100 μL of ddH_2_O was added to 100 μL of each thawed sample and vortexed for 5 min. The mixture was centrifuged at 10,000 rpm for 10 min at 4°C. The supernatant was then filtered by 0.22 μm membrane filtration and prepared for LC-MS analysis. To validate the reproducibility of the LC-MS system, 20 μL from prepared samples was pooled to generate the quality control (QC) samples.

### Chromatographic Separation and Mass Spectrometry Analysis

Chromatographic separation was accomplished in an Acquity UPLC system equipped with an ACQUITY UPLC® BEH C18 (100 mm × 2.1 mm, 1.7 μm, Waters) column maintained at 40°C. The temperature of the autosampler was 4°C. Gradient elution of analytes was carried out with 0.1% formic acid in water (A) and 0.1% formic acid in acetonitrile (B) at a flow rate of 0.25 mL/min. Ten microliter of each sample was injected after equilibration. An increasing linear gradient of solvent B (v/v) was used as follows: 0~1 min, 2% B; 1~9.5 min, 2~50% B; 9.5~14 min, 50~98% B; 14~15 min, 98% B; 15~15.5 min, 98~2% B; 15.5~17 min, 2% B.

The ESI-MSn experiments were executed on the Thermo LTQOrbitrap XL mass spectrometer with the spray voltage of 4.8 and −4.5 kV in positive and negative modes. Sheath gas and auxiliary gas were set at 45 and 15 arbitrary units. The capillary temperature was 325°C. The voltages of the capillary and tube were 35 and 50, −15 and −50 V in positive and negative modes. The Orbitrap analyzer scanned over a mass range of m/z 50–1,000 for full scan at a mass resolution of 60,000. Data dependent acquisition (DDA) MS/MS experiments were performed with the CID scan. The normalized collision energy was 30 eV. Dynamic exclusion was implemented with a repeat count of 2, and exclusion duration of 15 s.

### Data Processing and Analysis

The initial analysis examined the participant demographics of the two groups with the Statistical Package for the Social Science version 19.0 (SPSS Inc., Chicago, Illinois) and the GraphPad Prism version 5.0 (GraphPad Software Inc., San Diego, CA). Continuous data were checked for normal distribution using the Shapiro–Wilk test first. For normally distributed data, the independent two-sample *t*-tests were used to compare the means of two groups. For those data that were not normally distributed, non-parametric tests (Mann–Whitney *U*-test) were used for unpaired comparison between groups.

Urine metabolomic data from UPLC/MS were standardized to eliminate urine volume variability. Multivariate statistics techniques were used to analyze the multiple variables simultaneously in order to extract comprehensive information from the large metabolomics data and to subsequently visualize and interpret it. Comprehensive and integrative metabolomic data was mainly analyzed using metaboanalyst 4.0 (https://www.metaboanalyst.ca/) (24). Metabolomic data was first normalized by a pooled sample from the HC group and then log transformation and auto scaling were used during data processing. Principal component analysis (PCA), which was considered as one of the most widely used unsupervised techniques for the analysis of metabolomics data, was used for an initial exploration of the data mainly to obtain an overview and reveal clustering or patterns in the data (24, 25). Additionally, partial least-squares-discriminant analysis (PLS-DA), which was a typical supervised technique applied to metabolomics data, was used to construct a model which separated the different groups of samples on the basis of their metabolite features (25, 26). The variable importance in the projection (VIP) scores were calculated to reflect the importance of metabolite features in the model, and features with a VIP ≥ 1.0 and *p* ≤ 0.05 were selected as discriminating metabolites between groups (16, 27). The discriminating metabolites identification and metabolic pathway analysis were carried out using the methods described in previous studies (28, 29). To identify models with good performance, an algorithm based on Monte-Carlo cross validation (MCCV) coupled with the well-established algorithm Support Vector Machine (SVM) were used following the procedure as described in the protocol (14, 24). The discriminatory power was quantified by multivariate receiver operator curve (ROC) analysis calculating the area under the curve (AUC), sensitivity, specificity, and accuracy using the MetaboAnalyst 4.0 (24). A value of *p* < 0.05 (two-tailed) was considered statistically significant, and for multiple comparisons, the Benjamini–Hochberg procedure was used for controlling the false discovery rate (FDR).

## Results

### Participants Characteristics

A total of 60 boys (30 in ASD, 30 in HC group) met the inclusion criteria and were enrolled in this study. Most of the participants (98.3%) were Han Chinese, with one health control (1.67%) from Hui Minority living in China. Descriptive statistics for baseline characteristics are presented in Table 1. The two groups were well-matched for chronological age in a relative narrow range (age range: 2.6~6.7 years). The sample size allowed a statistical power of 0.85 for plasma and urine analysis (software G^*^Power, estimation for Wilcoxon–Mann–Whitney test, two tails, effect size *d* = 0.8, α = 0.05). All the children in the ASD group completed the CARS and ADOS assessments, and 29 of them completed the ADI-R assessment. Twenty-three autistic children and 22 HC children completed the GDS assessment. As expected, autistic children got significantly lower scores in the GDS than HC children (*p* < 0.001).

**Table 1:** Participant demographics.

	**ASD**	**HC**
*n*	30	30
Age, month	55.45 ± 2.357	53.01 ± 1.679
**GDS**
Adaption^1^	59.83 ± 3.648	99.27 ± 2.229
Gross motor^1^	70.91 ± 2.763	108.5 ± 2.282
Fine motor^1^	67.87 ± 3.660	96.27 ± 1.480
Language function^1^	50.00 ± 3.450	98.41 ± 2.575
Personal/social function^1^	62.70 ± 3.103	103.1 ± 3.231
**CARS**	35.20 ± 0.7530	
**ADOS**
Communication	5.433 ± 0.2233	
Social interaction	9.433 ± 0.2699	
Play/imaginative use of materials	2.067 ± 0.2141	
Restricted and repetitive behaviors	2.200 ± 0.1819	
**ADI-R**
Qualities of reciprocal social interaction	19.41 ± 0.9807	
Communication and language	14.76 ± 0.8059	
Restricted and repetitive, stereotyped interests and behaviors	5.966 ± 0.7166	

*GDS, Gesell Developmental Schedules; CARS, Childhood Autism Rating Scale; ADOS, Autism Diagnostic Observation Schedule; ADI-R, Autism Diagnostic Interview-Revised*.

*Data are presented as mean ± SEM unless otherwise indicated*.

1 *p < 0.001*.

### Metabolic Profiling Using Untargeted Metabolomics in LC-MS Platform

Plasma and urine metabolic profiling was established in an LC-MS platform to explore metabolic patterns associated with ASD. A total of 2,911 and 3,484 precursor molecules were detected in the positive and negative mode in the plasma samples, and 2,723 and 3,704 precursor molecules were detected in the positive and negative mode in the urine samples, respectively.

**Figure 1:**
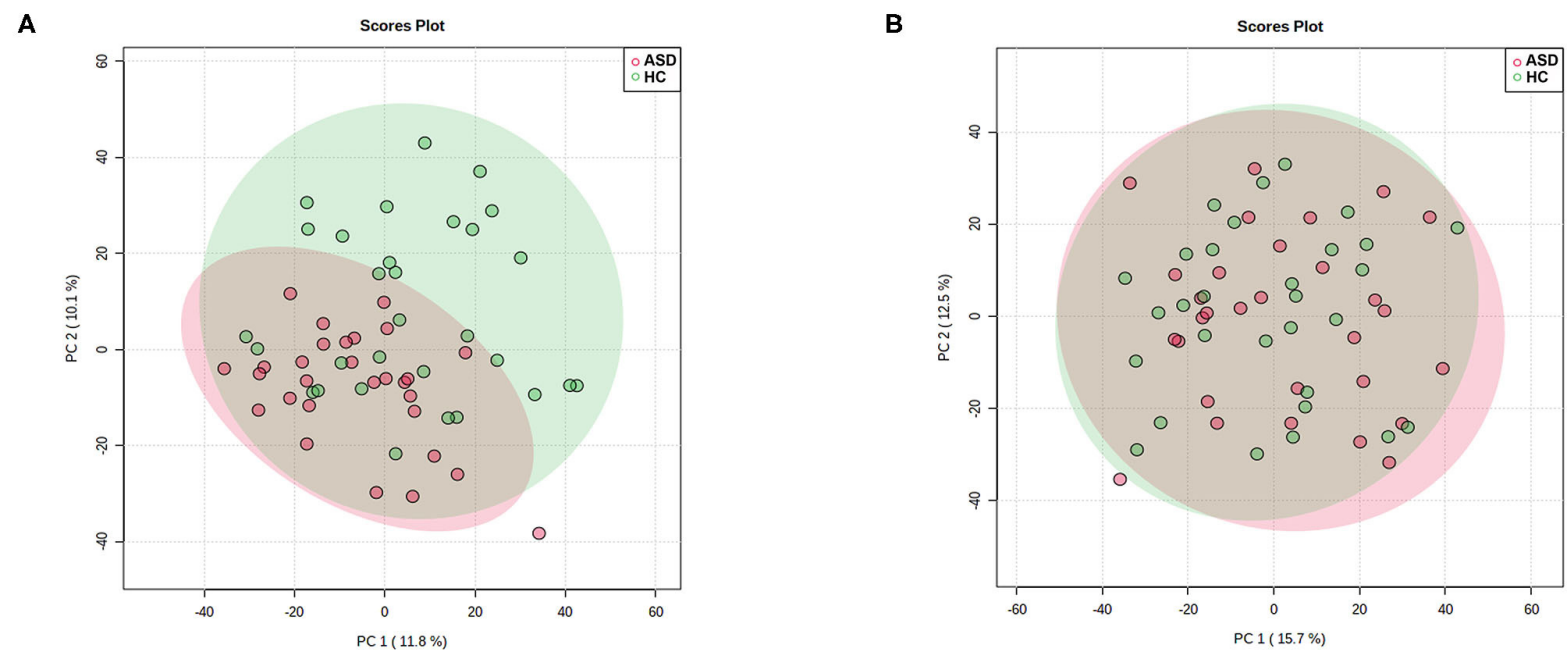
Principal Component Analysis (PCA) score plot in ASD (red) and HC (green) boys. Each point represents the metabolome score of a single individual. **(A)** Plasma, positive mode; **(B)** Urine, positive mode. The shaded areas indicate the 95%confidence ellipse regions for each group.

As is shown in Figure 1 PCA score plot, when labeling the samples as ASD and HC, no significant patterns of clustering could be detected in either the urine or plasma samples, suggesting that it was not possible to identify a valid separation using this approach. However, it seems that the ASD samples were more concentrated as compare to the scattered pattern of HC samples in plasma.

### Discriminating Metabolites and Multivariate Exploratory Analysis

**Figure 2:**
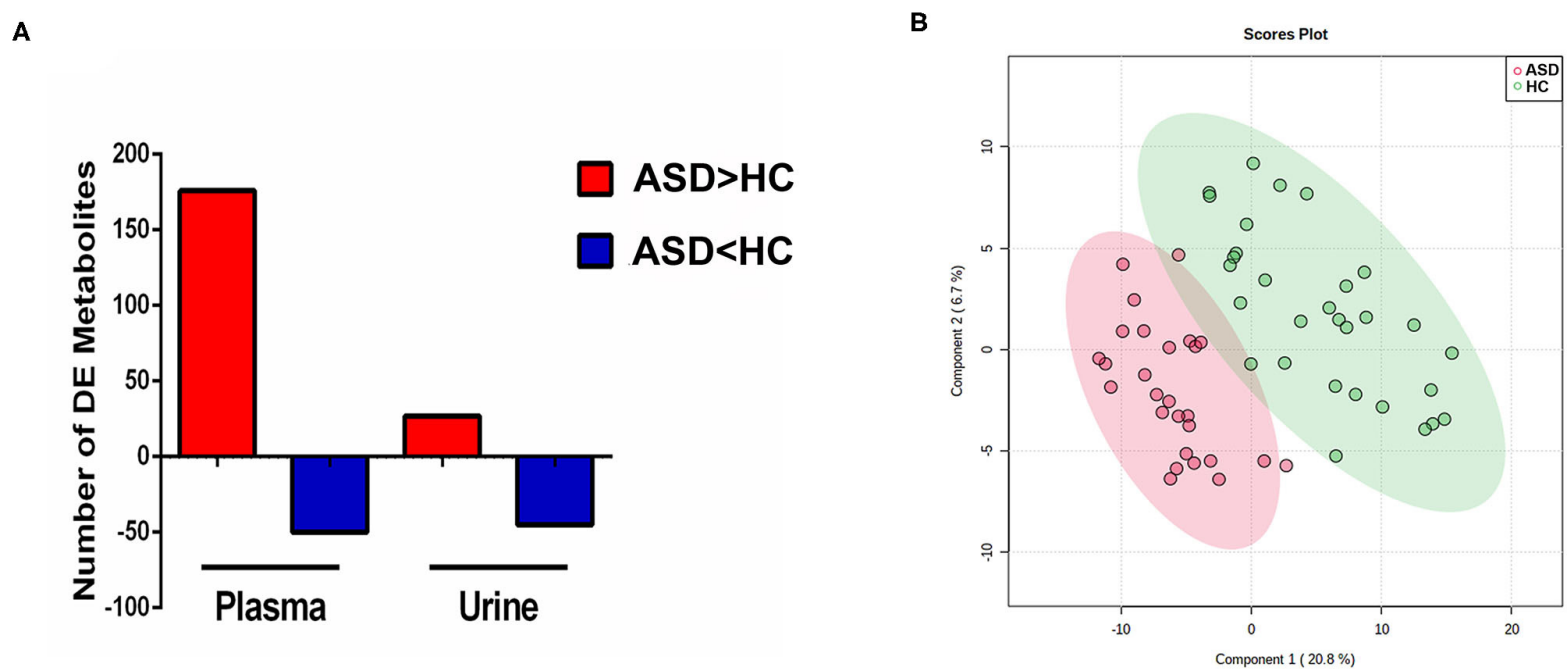
Discriminating metabolites and partial least squares discrimination analysis (PLS-DA). **(A)** Number of discriminating (DE) metabolites between groups in plasma and urine samples. **(B)** Partial least squares discrimination analysis (PLS-DA) score plot in ASD (red) and HC (blue) boys. Each point represents the metabolome score of a single individual. The shaded areas indicate the 95%confidence ellipse regions for each group.

The discriminating metabolites were screened with *p* ≤ 0.05 and VIP ≥ 1. When comparing the data of the ASD with HC group as detected in the positive mode, it is revealed that levels of 176 metabolites were higher and 50 metabolites were lower in the plasma samples, while levels of 27 and 45 metabolites were shown to be higher and lower in the urine samples (Figure 2A). As demonstrated in Figure 2B, the 2D scores plot constructed using partial least squares discrimination analysis (PLS-DA) with these discriminating metabolites, revealed good separation between ASD and HC groups (*R*^2^ = 0.816, *Q*^2^ = 0.624, and Accuracy = 0.897).

### The Discriminating Metabolites Identification and Biomarker Analysis

**Figure 3:**
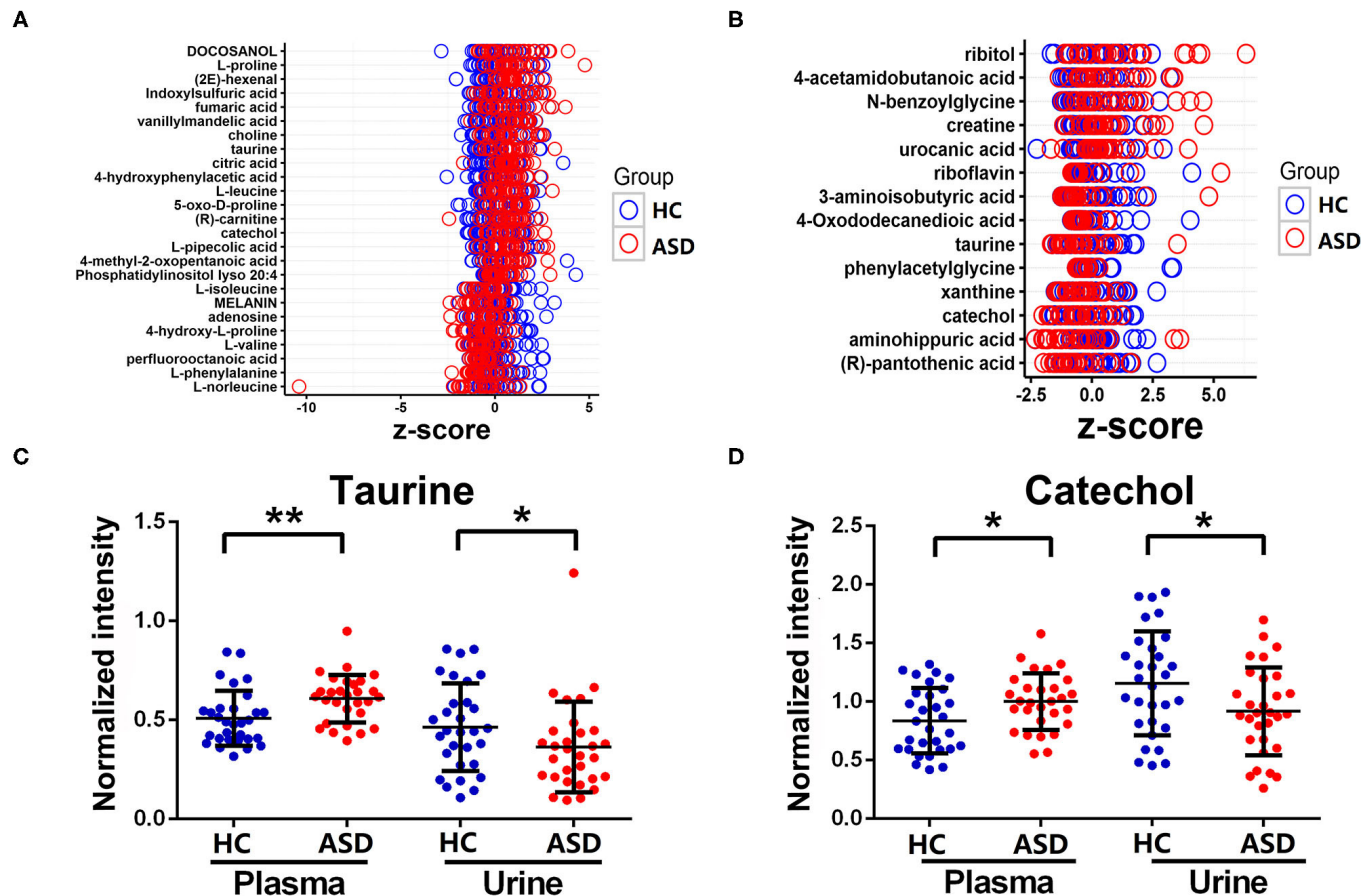
The discriminating metabolites differ between ASD and HC groups. **(A,B)** *Z*-scores of the discriminating metabolites were plotted in plasma and urine samples. **(C,D)** Levels of taurine and catechol in plasma and urine samples. Data are presented as mean ± SD, *p < 0.05, **p < 0.01.

A total of 25 and 14 discriminating metabolites were identified in plasma and urine samples, respectively. The *Z*-scores of the discriminating metabolites associated with ASD were demonstrated in Figure 3. Among them, taurine and catechol were found to be discriminating metabolites in both of the plasma and urine samples. However, levels of taurine and catechol were both found to be higher in plasma samples while lower in urine samples in the ASD as compare to the HC group. The correlation matrices of these discriminating metabolites were demonstrated in Supplementary Figure 1.

**Figure 4:**
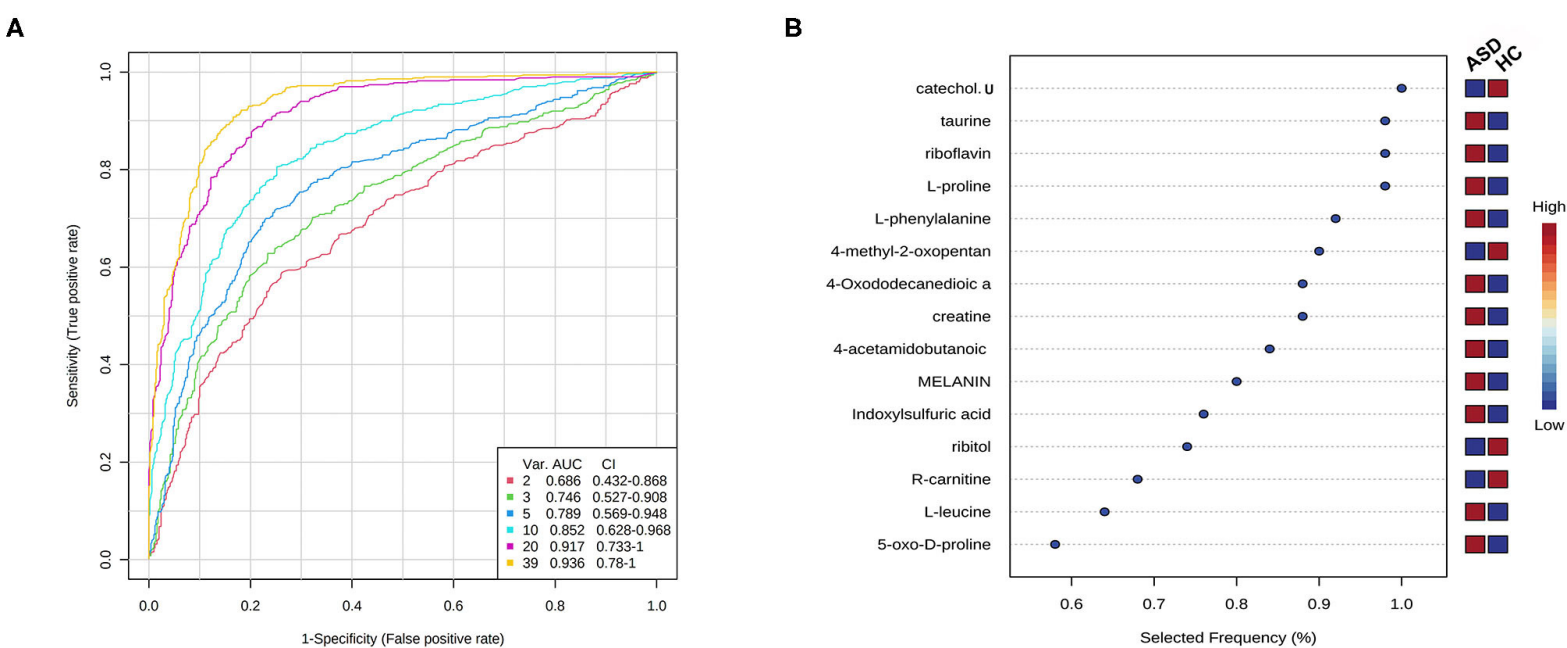
Potential biomarker analysis using SVM algorithm. **(A)** ROC curves from different biomarker models using different numbers of features. **(B)** The top 10 significant metabolites ranked based on their frequencies of being selected during cross validation.

The algorithm of SVM was used to perform potential biomarker analysis together with MCCV through balanced subsampling to identify models with good performance. Based on the cross validation, the multivariate biomarker models using 10 discriminating metabolites achieved an AUC of 0.852 (Figure 4A) with sensitivity of 0.833, specificity of 0.800 and accuracy of 0.817 (Supplementary Table 1). The top 10 significant metabolites ranked based on their frequencies of being selected during cross validation in the models are listed in Figure 4B.

### Metabolic Pathway and Function Analysis

**Figure 5:**
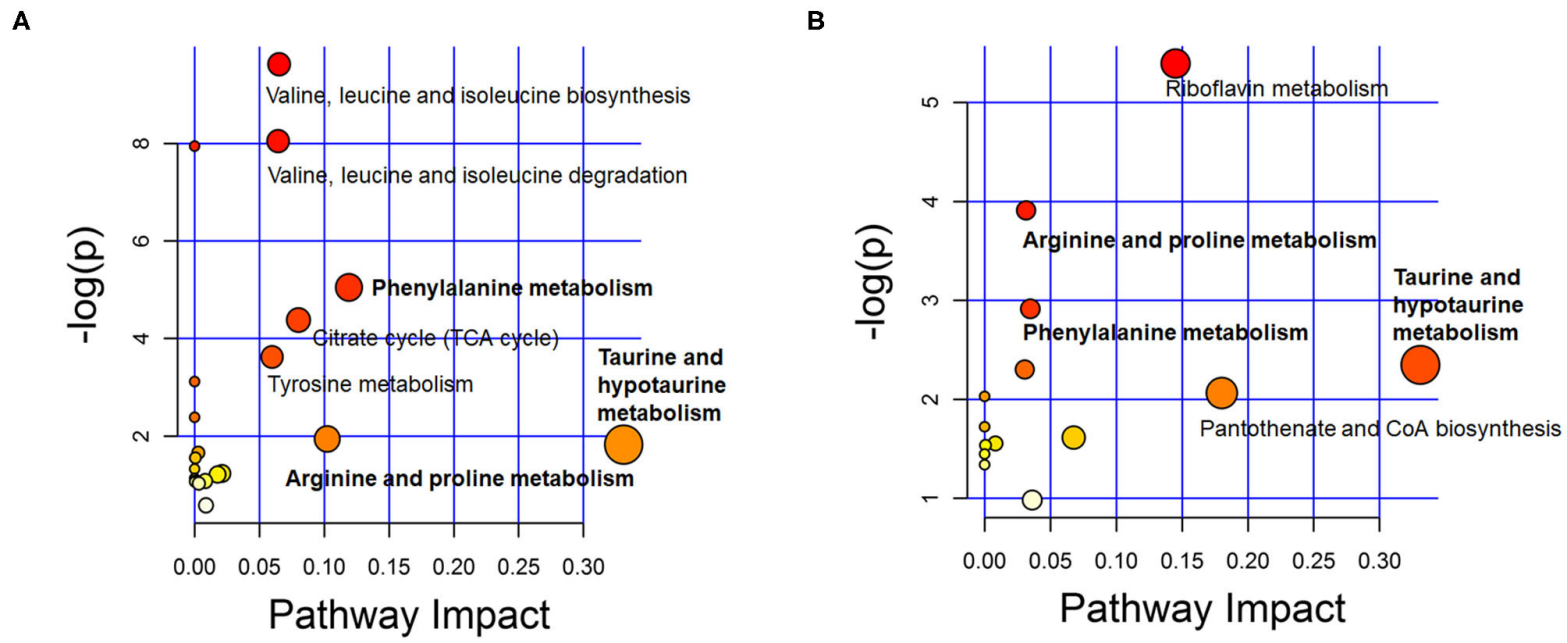
Overview of metabolic pathway analysis plot with MetPA in plasma **(A)** and urine **(B)**. Color intensity (white to red) reflects increasing statistical significance, while circle diameter covaries with pathway impact.

**Figure 6:**
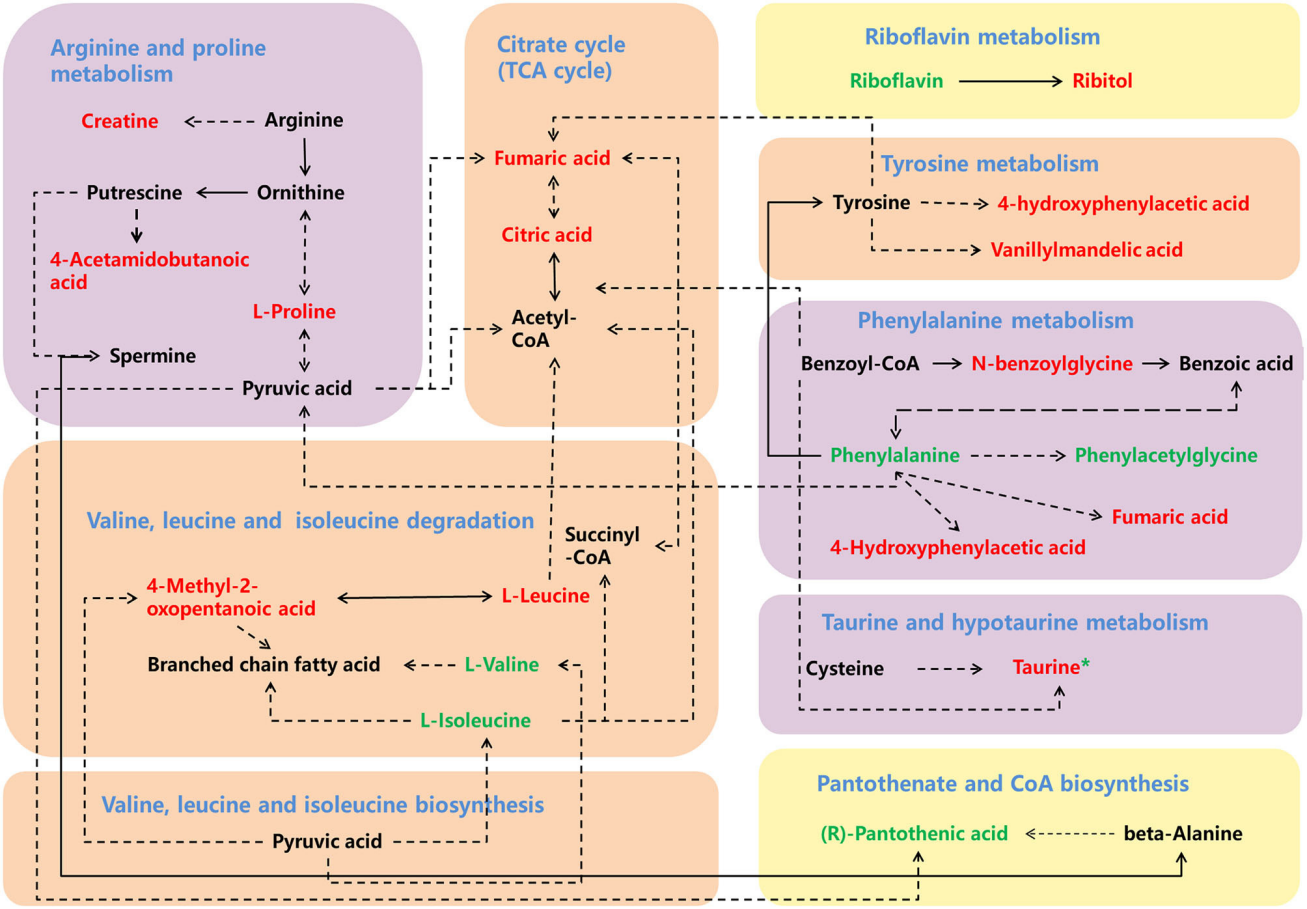
A schema showing the key enriched metabolic pathways implicated in ASD. The increased discriminating metabolites are labeled in red, and decreased discriminating metabolites are labeled in green, while the green * labeled taurine indicates its decreased level in urine. The yellow, orange, and purple backgrounds indicate enriched metabolic pathways in urine, plasma and both urine and plasma samples.

To identify the metabolic pathways associated with ASD, the discriminating metabolites were introduced in the pathways enrichment analysis. Results of the enriched pathway analysis using data from plasma samples revealed that the most perturbed metabolic pathway in ASD mainly corresponded to taurine and hypotaurine metabolism, phenylalanine metabolism, arginine and proline metabolism, valine, leucine and isoleucine biosynthesis and degradation. While the metabolic pathways corresponding to taurine and hypotaurine metabolism, pantothenate and CoA biosynthesis, riboflavin metabolism, phenylalanine metabolism, and arginine and proline metabolism were revealed to be perturbed in the ASD urine samples (Figure 5). The key enriched metabolic pathways implicated in ASD are summarized in Figure 6.

## Discussion

Metabolomics is the study of substrates and products of metabolism, which provides a tool to compare the profiles of small-molecule metabolites and identify their perturbations in metabolic pathways (30, 31). In the present study, levels of 176 and 27 metabolites were found to be higher, while 50 and 45 metabolites were lower in the positive mode in the plasma and urine samples of the ASD children, respectively. Notably, taurine and catechol levels were decreased in urine but increased in plasma in the same cohort of ASD children as compared to HC children. Results of the enriched pathway analysis using discriminating metabolites revealed that the most perturbed metabolic pathway in ASD mainly correspond to taurine and hypotaurine metabolism, phenylalanine metabolism, arginine and proline metabolism, pantothenate and CoA biosynthesis, and riboflavin metabolism.

Since metabolite profiling is sensitive to both genetic and environmental factors, it is considered as a powerful investigative approach with immense biomedical potential which could provide a multifactorial overview of an individual's status (32). Meanwhile, because it is sensitive to many influence factors, it is of great importance to control these confounders, such as age, gender, dietary status and drug intake (33–36). In this study, we strictly controlled these potential confounders to explore meaningful metabolic changes associated with ASD. As a dimensionality-reduction technique, PCA provided a full picture overview of the metabolic characters without artificial interference (24, 25). It seems that the ASD group largely overlapped with HC groups, especially in urine samples. But compared with the scattered distribution pattern in urine samples, the plasma samples of ASD only overlapped with part of the HC group, which suggested that compared with the diversity found in the HC group, children in the ASD group may share more similarities in metabolic patterns. And further analysis of these alterations in metabolic patterns will provide important information for the understanding of the pathogenesis of ASD. Additionally, more discriminating metabolites could be found in plasma than in urine. A relative good separation between the ASD and HC groups was achieved using PLS-DA with these discriminating metabolites. However, as a supervised classification method, the PLS-DA model may be over-fitting to some extent (37).

When comparing the results of the metabolic perturbations in plasma and urine associated with ASD, we found that two discriminating metabolites (taurine and catechol) were identified in both of the plasma and urine samples. As one of the most common amino acids in the brain, taurine can act as a neuromodulator to regulate the balance of excitatory and inhibitory neuronal activity (38, 39). Additionally, it has also been suggested that taurine has many positive effects such as an antioxidant, anti-inflammatory, gut-regulatory and immune modulator which may alleviate some symptoms associated with ASD (38, 40–42). Perturbed taurine levels have been found to be associated with ASD in a number of previous studies (43–48). However, findings regarding the plasma and urine levels of taurine are controversial (40, 43–50). Our results demonstrated that taurine levels were decreased in urine but increased in plasma in ASD children, which is consistent with some of the previous studies (43, 44, 46, 48). The opposite trend found in the urine and plasma samples from ASD children in the same cohort indicated that levels of taurine in the urine and plasma were not parallel, which suggest that the altered metabolic patterns associated with ASD are very complex, and levels of some metabolites in urine may not mirror their levels in plasma. So we cannot infer ASD children are lack of some nutrient substances only based on the lower levels of related metabolites found in urine samples. The importance to have a detailed analysis of the metabolites in different biological samples should be emphasized. Moreover, although there is a consistent opinion that taurine plays a protective role in ASD, and it has been proposed that the elevated plasma taurine levels found in ASD is compensatory against pathogenesis of ASD (such as oxidative stress) (48, 51, 52), the biological significance of taurine in pathogenesis of ASD still needs to be further studied.

Catechol, also known as pyrocatechol and 1,2-dihydroxybenzene, is a naturally occurring and an important industrial chemical (53). Metabolism of catechol involves several enzymes including Catechol-O-methyltransferase (COMT), which catalyzes the O-methylation of various endogenous and exogenous catecholic substrates (54, 55). It has been suggested that altered methylation metabolism of endogenous catechols due to COMT polymorphism may be a risk factor for the development of certain neurodegenerative disorders, as well as ASD (55–59). Additionally, this catechol-metabolizing system may be affected by exogenous factors such as catechol-containing polyphenols, which provide a feedback inhibition of methylation of endogenous catechols *in vivo* (54, 60). For example, the large amount of dietary catechol and catecholic polyphenols in organ oil or tea may collectively inhibit the methylation of endogenous bioactive catechols (54, 60, 61). Moreover, there is an emerging body of evidences demonstrating alterations in the gut microbiota composition between children with ASD and controls (62–64). Changes of catechol levels may also be related to alterations in gut microbiota, since gut microorganisms play critical roles in the production of catecholic substrates in gut lumen (65, 66), and these biologically active catecholic substrates may also affect the growth rates of some pathogenic bacteria (67, 68). Endogenous catechols in plasma were found to be abnormal in some autistic individuals and levels of catechols and their metabolites were found to be decreased in urine samples in ASD (59, 69). However, only plasma or urine samples were collected and measured in these previous studies. It is noteworthy that the decrease in urine catecholic substrate might accompany an increase in plasma, as observed in another study that simultaneously measured both plasma and urine samples from patients diagnosed with Alzheimer's disease (70). In this study, levels of catechol as well as taurine were also found lower in urine but higher in plasma in the same cohort of ASD children. As suggested by Fonteh et al. (70), the opposite trend in plasma and urine may be related to the facts that plasma contents are regulated by metabolic processes controlling the absorption, transport, degradation, and excretion of these molecules, while concentrations in urine will be influenced by the rate of excretion and reabsorption of these molecules. Certainly, further studies are required to determine the mechanisms behind these phenomena. High levels of catechol and its related metabolites could affect the activity of neurons and were found to be associated with behavior disturbances in ASD and other neuropsychiatric disorders (71–73). In this study, the higher levels in plasma in the ASD group indicated that catechol may cause some adverse health effects. Further studies are necessary to clarify the biological role of catechol in ASD.

Although different discriminating metabolites were found in plasma and urine samples, results of the metabolic pathway analysis revealed that perturbations in pathways corresponding to taurine and hypotaurine metabolism, phenylalanine metabolism and arginine and proline metabolism could be found in both of the plasma and urine samples. The association of ASD and phenylketonuria (PKU), a disease induced by deficiency in the metabolism of phenylalanine, has been well-documented, and children with PKU often show some autistic-like behavior (74–76). Significant differences in urine and plasma levels of phenylalanine have also been found to be associated with ASD in several independent previously published studies (9, 77–82), although some of these results were contradictory, all their data points to the perturbation of phenylalanine metabolism in ASD. Our results further confirmed this association, as perturbed phenylalanine metabolism in ASD was found in both of the metabolic pathway analysis results from the urine and plasma samples. Further studies are warranted to assess the diagnostic value of perturbation in the phenylalanine metabolism, as well as its therapeutic potential (83).

For the arginine and proline metabolism, decreased renal clearance of arginine has been found in ASD which indicated increased arginine transporter activity, and results of research on velocardiofacial (22q11.2 deletion) syndrome suggest that individuals with elevated plasma proline levels and the *COMT*^*MET*^ genotype were more likely to present with severe autism symptomatology (84). It has been suggested that proline plays a role in the modulation of brain function including its regulation of the basal function of some glutamate synapses, and abnormally elevated levels of proline in plasma were found to be associated with higher incidence of seizures and intellectual disability in hyperprolinemias, which may be related to a proline-induced reduction in glutamate release (85–87). Reduced glutamate release of the cerebral cortex has been found in the mouse model of ASD (88, 89) and abnormal glutamate concentrations in the brain have also been reported in individuals with ASD (90, 91).

The strength of our study includes: (1) To our knowledge, this is the first study to collectively analyze the untargeted metabolic profiles from both of the plasma and urine samples obtained at the same time point from the same cohort of ASD children, which gave us the opportunity to see a more comprehensive picture of the full metabolic status associated with ASD; (2) Diagnosis of ASD was not only made according to the DSM-IV criteria, but also confirmed with ADOS and ADI-R, which were considered as the gold standard; (3) The participants involved in this study were only boys and were tightly matched in a relatively narrow age range to minimize the potential confounding effects associated with gender and age. However, as a preliminary study, there are also several limitations which ought to be mentioned: (1) The sample size in this study is relatively small, which may decrease the statistical power; (2) There was no validation group in this study to test the results in an independent cohort; (3) Participants in this study were mainly from north China and most of them were from the Han population, and the narrow age range may affect the external validation of the results; (4) Although all the samples were collected after an overnight fasting to minimize the potential effects of food and water intake, variations in children's recent dietary preferences were not strictly controlled in this study. These limitations should be considered when data are interpreted.

These preliminary results, despite the sample size, identified substantial biochemical differences and several metabolic pathways associated with ASD, which may improve our understanding of the biochemical mechanisms of ASD. Further targeted analyses in a larger cohort are needed to validate these preliminary results in this study. Additionally, the ROC curve results have the potential to contribute to the diagnosis of ASD, but merit further investigations in larger cohorts especially among other developmental disorders to assess its specificity and to screen for potential ASD-specific biomarkers. And results from these studies, together with other systems biology approaches such as transcriptomics would also provide new clues for the individualized biomedical treatment for ASD.

In summary, results of the present study suggest that a series of common perturbations in the metabolic profile may exist in children with ASD, which are possibly associated with genetic or epigenetic variations in metabolic enzymes, gastrointestinal dysfunctions, and nutrient deficiency. It also highlights the importance of having a comprehensive analysis of the metabolites in different biological samples to reveal the full picture of the complex metabolic patterns associated with ASD. These results and further related studies would provide new clues for study of the mechanism and systemic biomedical treatment for ASD.

## Data Availability Statement

The raw data supporting the conclusions of this article will be made available by the authors, without undue reservation.

## Ethics Statement

The studies involving human participants were reviewed and approved by Peking University Institutional Review Board. Written informed consent to participate in this study was provided by the participants' legal guardian/next of kin.

## Author Contributions

X-JX, XY, and RZ: conceptualization. X-JX, F-CM, T-JS, and BL: methodology. X-EC and BL: validation. X-JX, JW, and X-EC: formal analysis. XJ-X, X-EC, F-CM, and X-XW: investigation. X-JX and BL: writing—original draft preparation. Y-ZW, JW, and RZ: writing—review and editing. Y-ZW and F-JZ: visualization. XY and RZ: supervision. X-JX and RZ: project administration and funding acquisition. All authors contributed to and approved the final manuscript.

## Conflict of Interest

The authors declare that the research was conducted in the absence of any commercial or financial relationships that could be construed as a potential conflict of interest.

## Abbreviations

ADI-R: Autism Diagnostic Interview-Revised
ADOS: Autism Diagnostic Observation Schedule
ASD: Autism spectrum disorder
AUC: Area under curve
CARS: Childhood Autism Rating Scale
DDA: Data dependent acquisition
FDR: False discovery rate
GC-MS: Gas chromatography-mass spectrometry
GDS: Gesell Developmental Schedules
HC: Healthy control
LC-MS: Liquid chromatography-mass spectrometry
MCCV: Monte-Carlo cross validation
NMR: Nuclear magnetic resonance
PCA: Principal component analysis
PKU: Phenylketonuria
PLS-DA: Partial least squares discrimination analysis
ROC: Receiver operating characteristic
SVM: Support Vector Machine
UPLC: Ultra performance liquid chromatography
VIP: Variable importance in the projection.
