# Supplementary material for: Comparison of the Metabolic Profiles in the Plasma and Urine Samples Between Autistic and Typically Developing Boys: A Preliminary Study
Source: Front Psychiatry. 2021 Jun 4;12:657105. doi: 10.3389/fpsyt.2021.657105 (PMC8211775; doi:10.3389/fpsyt.2021.657105)
Supplement: Supplementary file 1 [file Data_Sheet_1.PDF]

# Supplementary Material

## Supplementary Figures

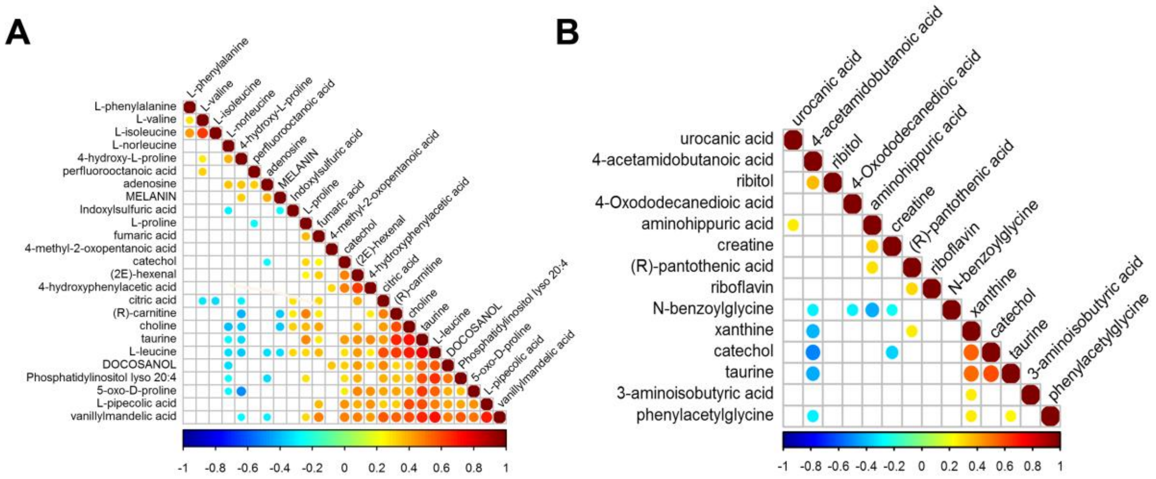


**Supplementary Figure 1.** The correlation matrices of discriminating metabolites in plasma **(A)** and urine **(B)**.
