# Supplementary material for: Comparison of the Metabolic Profiles in the Plasma and Urine Samples Between Autistic and Typically Developing Boys: A Preliminary Study
Source: Front Psychiatry. 2021 Jun 4;12:657105. doi: 10.3389/fpsyt.2021.657105 (PMC8211775; doi:10.3389/fpsyt.2021.657105)
Supplement: Supplementary file 2 [file Table_1.DOCX]

**TABLE S1 |** Summary of classifier performance metrics of the multivariate biomarker models.

| **Feature No.** | **Sensitivity** | **Specificity** | **Accuracy** | **AUC** |
| --- | --- | --- | --- | --- |
| 2 | 0.733 | 0.767 | 0.750 | 0.686 |
| 3 | 0.733 | 0.800 | 0.767 | 0.746 |
| 5 | 0.700 | 0.767 | 0.733 | 0.789 |
| 10 | 0.833 | 0.800 | 0.817 | 0.852 |
| 20 | 0.900 | 0.833 | 0.867 | 0.917 |
| 39 | 0.933 | 0.933 | 0.933 | 0.936 |
